# Elevated core temperature in addition to mental fatigue impairs aerobic exercise capacity in highly trained athletes in the heat

**DOI:** 10.1186/s40101-024-00377-0

**Published:** 2024-11-25

**Authors:** Takashi Naito, Tatsuya Saito, Hirotsugu Morinaga, Nobuhiko Eda, Yohei Takai

**Affiliations:** 1 Faculty of Law, Hokkai-Gakuen University, 4-1-40 Asahi-machi Toyohira-Ku, Sapporo-City, Hokkaido 062-8605 Japan; 2 Faculty of Medicine, Tottori University, 4-101 Koyama-cho minami, Tottori-City, Tottori 683-8550 Japan; 3 Department of Sports and Life Science, National Institute of Fitness and Sports in Kanoya, 1 Shirouzucho, Kanoya-City, Kagoshima 891-2391 Japan; 4 Department of Fundamental Educaion, Dokkyo Medical University, 880 Kitakobayashi, Mibu, Shimotsugagun, Tochigi, 321-0293 Japan

## Abstract

The purpose of this study was to investigate the effects of elevated core temperature by exposure to heat stress vs. heat exposure without elevated core temperature (mean skin temperature only) in addition to mental fatigue on aerobic exercise capacity in the heat. Seven highly trained athletes completed two experimental conditions: elevation in core and skin temperatures (hyperthermia: HYP), and skin temperatures (SKIN). Participants performed the AX-Continuous Performance Task and Stroop Task to induce mental fatigue during a warm water immersion at 40 °C (HYP) and a passive seated heat exposure in a climatic chamber at 35 °C and 60% relative humidity (SKIN) for 45 min before exercise. Thereafter, participants performed running trial at 80% maximal oxygen uptake until voluntary exhaustion in the same chamber as the SKIN. Exercise time to exhaustion was significantly shorter in the HYP trial (538 ± 200 s) than in the SKIN trial (757 ± 324 s). Rectal temperature at the end of tasks in the HYP trial increased by 0.86 ± 0.26℃ and was significantly higher (37.69 ± 0.18℃) than that of the SKIN trial (36.96 ± 0.13℃), albeit no significant differences in mean skin temperature. Self-reported mental fatigue using visual analog scale was significantly higher after tasks in both trials, but no significant difference between trials was found. Throughout the trial, salivary cortisol concentration and perceptual responses were not affected by hyperthermia. This study demonstrated that a combination of high core temperature and mean skin temperature, and mental fatigue limit aerobic exercise capacity in highly trained athletes in hot environments compared with heat exposure without an elevation of core temperature.

## Introduction

Sports competitive events are often held in hot climate conditions, imposing additional stress on athletes and resulting in high body core temperature (Tc) [1, 2]. Previous studies have reported that the attainment of a high Tc is the main limiting factor inhibiting exercise performance, as evidenced by a reduced central nervous system drive to the skeletal muscle and other adverse effects, including cardiovascular strain and metabolic disturbances [3, 4]. During endurance exercise in a hot environment, high mean skin temperature (Tsk) above 35℃ are also known to cause early fatigue [5]. Therefore, the development of hyperthermia is associated with limitations to aerobic exercise performance. In addition, it has been noted that when external environmental factors, such as radiant heat [6] or air velocity [7], are combined with ambient temperature and relative humidity, the additive and synergistic effects further impair thermoregulatory responses and aerobic exercise capacity.

Regarding the effect of internal environmental factors such as psychological state on exercise-induced fatigue, it is known that prolonged mental exertion leading to mental fatigue reduces endurance exercise performance. Mental exertion refers to the engagement with a demanding cognitive task such as the AX-continuous performance task (AX-CPT) or Stroop task [8–10]. Marcora et al. [8] showed that performing 90-min cognitive task using AX-CPT in 18–22 ℃ air temperature impaired subsequent exercise tolerance. Smith et al. [10] investigated the effects of mental fatigue on soccer-specific physical and technical performance. Their findings demonstrated that the performance of a 30-min modified Stroop task prior to a Yo-Yo intermittent recovery test resulted in a reduction in running distance compared to the control trial. Barzegarpoor et al. [11] showed that performing a 45-min Stroop task during submaximal cycling exercise increased the rating of perceived exertion (RPE), but the physiological responses to exercise were unaffected by mental fatigue. In response to these results, a recent review of previous studies indicates that higher RPE induced by mental fatigue during a certain task may reduce endurance exercise performance [12, 13]. Furthermore, the elevation of RPE is observed more frequently in hot than in thermoneutral conditions, leading to reduced endurance exercise performance [14].

Recently, the combined effects of mental fatigue from a prolonged cognitive task and exposure to heat stress have been investigated as a possible additional negative effect [13]. Otani et al. [15] demonstrated that heat stress-induced increase in core and skin temperatures, using warm water immersion during cognitive tasks, reduced subsequent aerobic exercise capacity compared to mental fatigue alone. Van Cutsem et al. [16] reported that mild mental fatigue caused by a cognitive task performed disappears after 30 min, and subsequent self-paced cycling effort at 30℃ did not affect time trial performance. The results of these two studies suggest that the combined negative effects on endurance exercise performance are likely to be due to the degree of intensity of heat stress and mental fatigue. In the focus on exposure to heat stress, it is not clear whether the effects of elevation of Tc and Tsk (hyperthermia) or heat exposure without elevation of Tc (Tsk only) during a cognitive task performed induce impairment of exercise capacity. It is possible that simultaneous exposure to mental fatigue and heat exposure that increases Tc would attenuate aerobic exercise capacity. The purpose of this study was to investigate the effects of elevated core temperature by exposure to heat stress vs. heat exposure without elevated core temperature (Tsk only) in addition to mental fatigue on aerobic exercise capacity in the heat. We hypothesized that aerobic exercise capacity in hot environments would be impaired by a combination of high Tc and Tsk, and mental fatigue compared with high Tsk and mental fatigue.

## Methods

### Participants

Eight non-heat-acclimatized highly trained male athletes were recruited for this study. However, one participant dropped out due to heat-related illness in the experimental trial. Therefore, seven non-heat-acclimatized highly trained male athletes (age: 22 ± 1 years, height: 173.2 ± 2.4 cm, body mass [BM]: 67.11 ± 5.2 kg, maximal oxygen uptake [●VO_2_max]: 63.7 ± 7.1 mL/kg/min) were participated for this study. They were categorized according to “tier 3” as highly trained by a previous study [17]. All the participants were non-smokers and normotensive, had no known autonomic dysfunction or cardiovascular disease, and were not taking any medications. The study protocol was approved by the National Institute of Fitness and Sports in Kanoya Ethics Committee (Permission number: 22–1–14), and all participants provided their informed consent to participate prior to commencing the study. The study complied with the latest version of the Declaration of Helsinki and was conducted according to international standards.

### Preliminary measurements

In order to determine the ●VO_2_max, on their first visit to the laboratory, each participant performed a progressive exercise test by treadmill (h/p/cosmos para graphics; h/p/cosmos, Germany) at room temperature (25 °C and 50% relative humidity [RH]). Their height and BM were measured to the nearest 0.1 cm and 10 g, respectively (TBF-210; Tanita, Japan). The protocol consisted of progressive exercise beginning at 8 km/h for 3 min, followed by increments of 2 km/h every 3 min until 16 km/h. And then speed was increased by 2 km/h every 2 min until volitional exhaustion [18]. Respiratory gases were measured every 30 s during the test using a pre-calibrated automatic gas analyzer (AE-310 s; Minato Medical Science, Japan). Heart rate (HR) was monitored continuously via telemetry using an HR monitor (RC3 GPS, Polar, Finland). The test was considered to be valid if two of the following three criteria were met: 1) oxygen consumption reached a plateau, 2) HR remained within 5% of the predicted maximum (220–age), or 3) the respiratory exchange ratio was above 1.10 [18].

### Experimental protocol

**Fig. 1:**
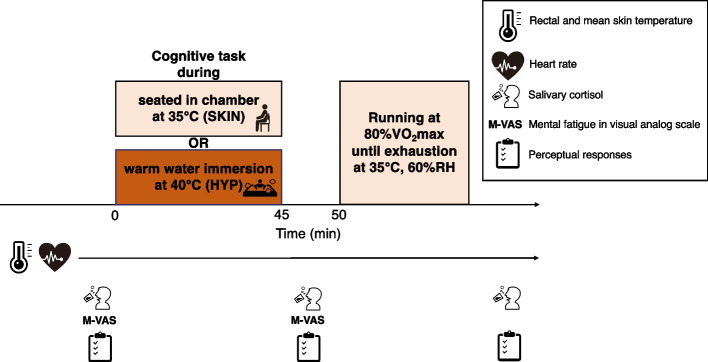
Schematic representation of the experimental protocol

The participants completed two trials in a randomized design: one with elevation in core and skin temperatures (hyperthermia: HYP), and another with just skin temperature (SKIN). All experimental trials were separated by at least 2 days to avoid an order effect and commenced at the same time to control for circadian variations in the core temperature. To avoid the influence of natural heat acclimatization all trials took place during the winter season. Throughout the study period, all participants were asked to keep their normal lifestyle activities at a stable level, including their physical activity and nutritional habits. During the 24-h period before the experimental trial, the participants were instructed to refrain from strenuous exercise and the consumption of alcohol, caffeine, and nutritional supplements. Each participant arrived at the laboratory after an overnight fast and refraining drinking any type of beverage for 2 h. Upon arrival at the laboratory, the participants had urine samples collected and were weighed. The participants were dressed in shorts and athletic shoes in all trials. The intrinsic clothing insulation value for this ensemble was 0.08 clo. [19] The participants were fitted with measuring equipment and asked to perform cognitive tasks, including the AX-CPT and Stroop task, for 45 min in order to induce mental fatigue. This was carried out while they were either immersed in warm water at 40 °C (HYP) or seated in a chamber at 35 °C with 60% RH (SKIN) for the same duration. Participants in the HYP trial were immersed to the upper chest at a seated posture in bathtub (1.5 × 1.0 × 0.5 m). The participants' perceptual responses and saliva samples were collected before and after the 45-min cognitive task. During the HYP trial, participants moved to the climatic chamber and performed the running exercise at an intensity equivalent to 80%●VO_2_max until reaching voluntary exhaustion (TTE) in the same hot environment as the SKIN trial. After the exercise, the participants dried themselves with a towel and were weighed again to determine their BM before the collection of saliva and urine samples (Fig. 1).

### Cognitive task

**Fig. 2:**
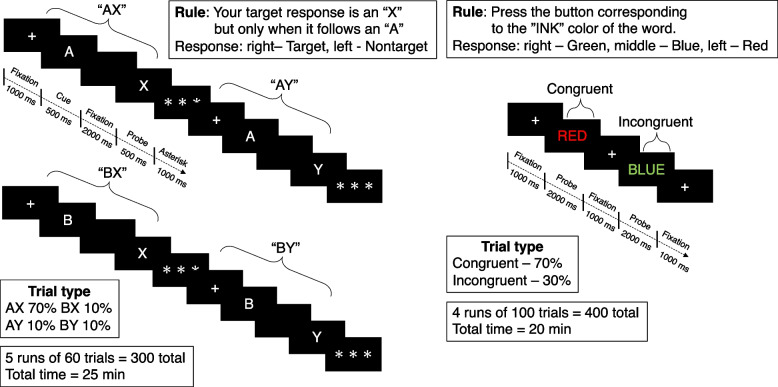
Schematic representation of the AX-CPT (**A**) and Stroop tasks (**B**) protocol

The 45-min task used as experimental manipulation in the present study is similar to those used by Lesh et al. [20]. Previous studies have demonstrated that the execution of the Stroop task for a duration of 30 to 45 min results in the induction of mental fatigue in physically active subjects [21, 22]. Recently, a previous study observed that the AX-CPT or Stroop task for a duration of 10 min also caused mental fatigue [23]. It is notable that the longer the task, the greater the mental fatigue. Accordingly, we attempted to enhance the mental fatigue for highly trained athletes by employing the 25-min AX-CPT (300 trials) and the 20-min Stroop task (400 trials), when the combination of fixation, cue and probe times for each task were taken into consideration. Task parameters and visual depictions of the AX-CPT and the Stroop task are presented in Fig. 2. Briefly, in the AX-CPT, participants make a target response (index finger button press) to the probe letter X only if it was preceded by the cue letter A. All cues and non-target probes require non-target responses (middle finger button press). Target sequence trials are frequent and set up a prepotent tendency to make a target response when the probe letter X occurs. As a result, non-target sequence trials where any non-A cue (collectively called B-cues) is presented and followed by a probe letter X, require the most cognitive control. The frequency of occurrence was 70% AX, 10% AY, 10% BX, and 10% BY, respectively.

In the Stroop task, stimuli consisted of one of three color words (RED, GREEN, and BLUE) that were written in one of three color inks (red, green, and blue). Stimuli could be Congruent (word and ink match) or Incongruent (word and ink do NOT match). Participants respond with a button press corresponding to the color of the ink of the word. The trials were arranged in pseudorandom sequence with 70% of trials being congruent (matched word and color), whereas 30% were incongruent, with all incongruent word–color combinations being equally common. Participants were instructed to respond as quickly and accurately as possible in both tasks. Cognitive task performance was assessed reaction time, lapses of attention, and accuracy by standard psychology software (Multi-PAS2, DKH, Japan). Attention lapses correspond to the number of trials with a reaction time longer than 2000 ms [24]. Response accuracy was the proportion of correct responses. The state of mental fatigue was characterized by an increase in reaction time, an increase in lapses of attention, and/or a decrease in accuracy [24]. A comparison of cognitive task performance was conducted between the initial and concluding 5 min of each task.

### Physiological and psychological measurements

#### Hydration status and body mass

Urine specific gravity (USG) was measured using a refractometer (PAL-09S, Atago, Japan) before and after exercise. Nude BM to the nearest 10 g was measured using a weighing scale (BC-316, Tanita, Japan) before and after the trial. Total sweating loss (TSL) was calculated using the following formula: *TSL* = *BM before the trial – BM after the trial*. Dehydration level was calculated using the following formula: *Dehydration level* = *TSL/ BM before the trial* × 100) [25].

#### Thermoregulatory responses

Throughout the two trials, Tre was obtained using a disposable rectal thermistor (ITP010-11, Nikkiso-Therm, Japan) that was self-inserted approximately 150 mm into the rectum. HR was monitored continuously via telemetry using an HR monitor (H10, Polar, USA) affixed to the chest. Four skin temperatures (chest, forearm, thigh, and calf) were also recorded via iButtons® (Thermocron SL type; KN Laboratory, Japan) affixed using hypoallergenic polyacrylate adhesive tape. The Tsk was calculated using the following formula from Ramanathan [26]: $Tsk\hspace{0.17em}=\hspace{0.17em}0.3\hspace{0.17em}\times \hspace{0.17em}(chest\, temperature)\hspace{0.17em}+\hspace{0.17em}0.3\hspace{0.17em}\times \hspace{0.17em}(upper\, arm \,temperature)\hspace{0.17em}+\hspace{0.17em}0.2\hspace{0.17em}\times \hspace{0.17em}(thigh\, temperature)\hspace{0.17em}+\hspace{0.17em}0.2\hspace{0.17em}\times \hspace{0.17em}(calf\, temperature)$. The core-to-skin temperature gradient was calculated using the following formula: *Core-to-skin temperature gradient* = *Tre – Tsk*.

#### Salivary cortisol

Saliva samples were collected as described previously both before and after the 45-min task and after exercise [27]. Participants sat and rinsed their mouths with distilled water for 30 s three times and then rested for 5 min. The participants were asked to expectorate their saliva through a short plastic straw into a collection vial. Subsequently, the saliva samples were frozen at − 80 °C and stored until the end of the study period. Cortisol concentration was measured using enzyme immunoassay kits (Salimetrics, Carlsbad, CA) according to the manufacturer’s protocol.

#### Perceptual scales

Self-reported mental fatigue was measured using a visual analog scale (M-VAS) before and after the 45-min task. Participants were asked to indicate their perceived level of mental fatigue (from not all to completely exhausted) by placing a mark on a 100-mm line [28].

Subjective thermal sensation (TS; 9-point scale ranging from 1 = “very cold” to 9 = “very hot”) [29] and comfort (TC; 7-point scale ranging from 1 = “very uncomfortable” to 7 = “very comfortable”) [30] were recorded at before and after both the 45-min task and TTE. The RPE (15-point scale) [31] was recorded before and after the TTE.

### Statistical analysis

Descriptive data were presented as means ± standard deviations. All statistical computations were performed using the IBM SPSS Statistics 28 software package (SPSS, Inc., USA). The normality of the data and homogeneity of variance between the trials were tested using Shapiro–Wilk’s and Levene’s tests, respectively. When the result of Shapiro–Wilk’s test or Levene’s test was less than the significant level, data were analyzed using non-parametric tests. Non-parametric data were analyzed using Friedman’s two-way analysis of variance (perceptual scales). When a significant difference was found, the pairwise comparisons were tested using Wilcoxon’s signed-rank test. In all other cases, a two-way (trial × time) repeated-measures analysis of variance was performed to compare the data for the different experimental conditions. Where appropriate, a paired sample t-test was then used to identify any changes. The TTE, TSL, and dehydration levels were compared using the dependent samples t-test. The level of significance was set at *p* < 0.05. Cohen’s d (d) was used as a measure of effect size for paired samples, with 0.2 to < 0.6, ≥ 0.6 to < 1.2, ≥ 1.2 to < 2.0, ≥ 2.0 to < 4.0, and ≥ 4.0 representing small, moderate, large, very large and extremely large treatment effects, respectively [32].

## Results

### Time to exhaustion

**Fig. 3:**
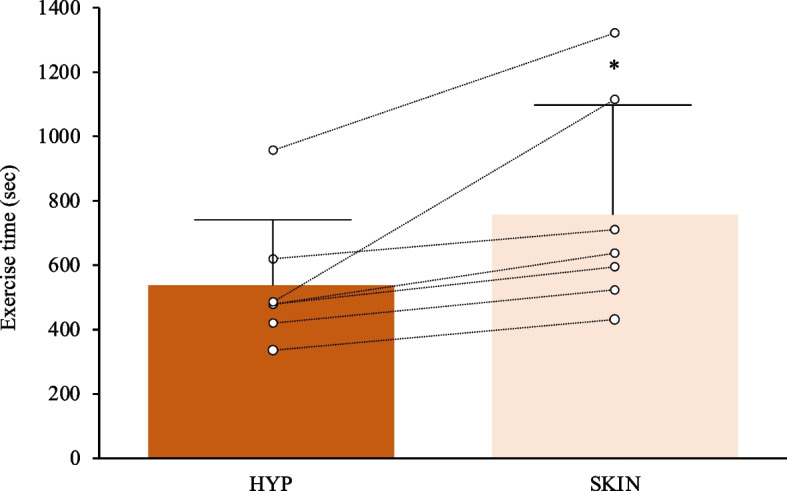
The time to exhaustion under the two experimental trials. The lines denote the raw data from individual participants (*n* = 7). *p < 0.05

All participants ran for a shorter time in the HYP trial than in the SKIN trial (*p* = 0.028, *d* = 1.12). The mean exercise time to exhaustion was 538 ± 200 s for the HYP trial, and 757 ± 324 s for the SKIN trial, respectively (Fig. 3).

### Thermoregulatory responses

**Fig. 4:**
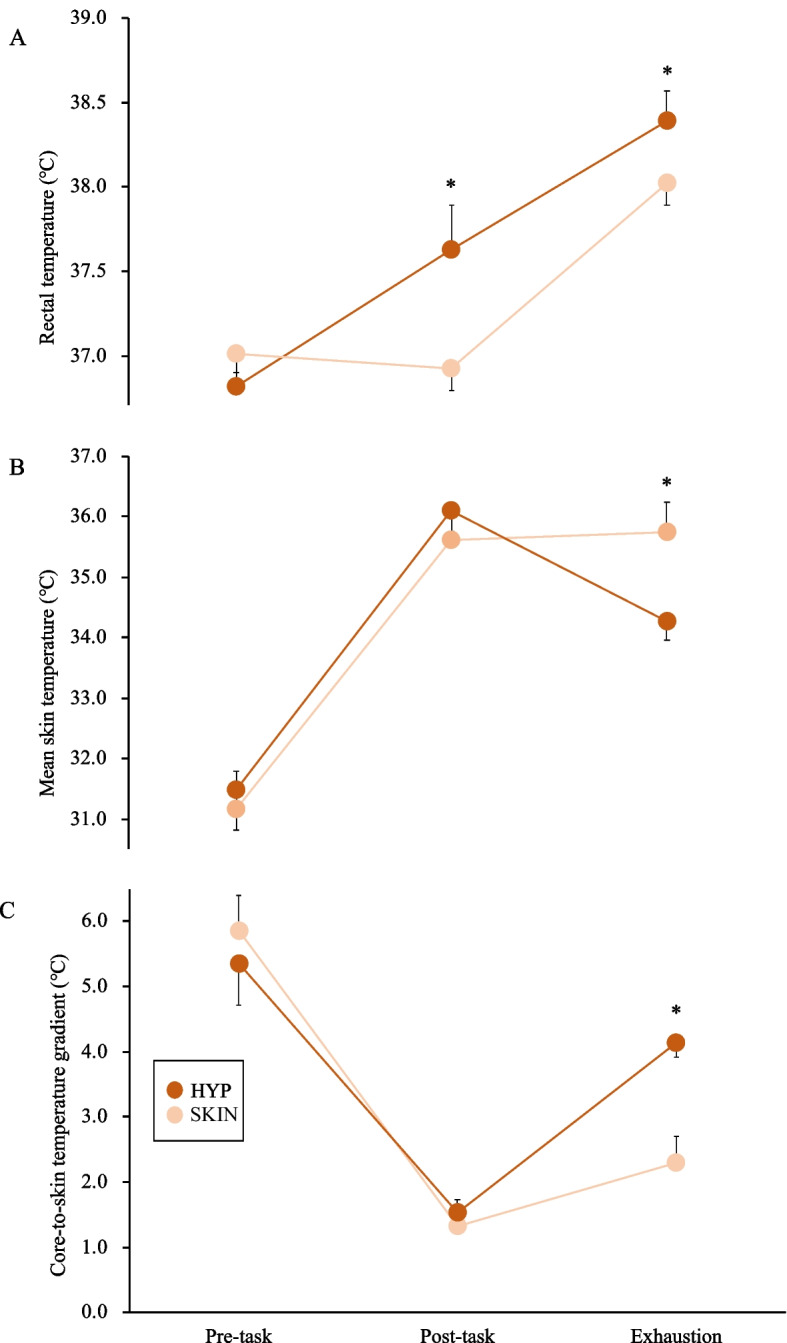
The rectal temperature (**A**), mean skin temperature (**B**), and core-skin temperatures gradient (**C**) under the two experimental trials. The mean values are expressed as mean ± SD. Time × intervention effect *p < 0.05, HYP vs. SKIN

At rest, Tre was similar between trials. A HYP trial increased Tre by 0.86 ± 0.26℃ (*p* = 0.001, *d* = 4.34) and was significantly higher (37.69 ± 0.18℃) than that of the SKIN trial (36.96 ± 0.13℃; *p* = 0.001, *d* = 4.65) at the end of task. The Tre increased progressively in both trials during exercise (Fig. 4A). Tsk increased progressively in both trials during the 45-min task, albeit there were significant differences in Tsk at the end of the task between trials. The Tsk at exhaustion was significantly lower in the HYP trial (34.86 ± 0.50℃) than in the SKIN trial (35.62 ± 0.29℃; *p* = 0.020, *d* = 1.86) (Fig. 4B). There were no significant differences in core-to-skin temperature gradient before and after the 45-min task (p > 0.05). The core-to-skin temperature gradient at exhaustion was significantly wider in the HYP trial (4.13 ± 0.37℃) than in the SKIN trial (2.28 ± 0.32℃; *p* = 0.001, *d* = 4.49) (Fig. 4C).

### Cognitive task performance, perceptual scales, and salivary cortisol

**Fig. 5:**
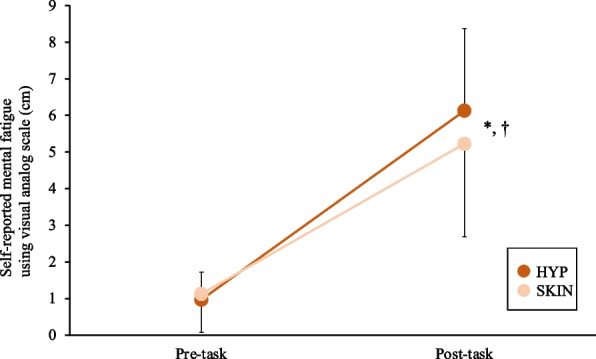
The self-reported mental fatigue using visual analog scale under the two experimental trials. The mean values are expressed as mean ± SD. *p < 0.05, vs. rest in the HYP trial. †*p* < 0.05 vs. rest in the SKIN trial

There were no significant differences in reaction time, lapses of attention, and accuracy for each task between trials (Table 1; *p* > 0.05). M-VAS increased progressively in both trials after the 45-min task, albeit no significant differences in M-VAS between trials (Figure 5; *p* = 0.349, *d* = 0.38). There was no significant difference in TS, TC, RPE, and salivary cortisol throughout the trials (Table 1).

**Table 1:** Cognitive task performance, perceptual scales, salivary cortisol, and heart rate in the experimental trial. Values are expressed as means ± SD (*n* = 7)

	Trial	Pre-task	First 5 min of AX-CPT	Last 5 min of AX-CPT	First 5 min of Stroop task	Last 5 min of Stroop task	Post-task	Exhaustion
Reaction time (ms)	HYP	-	0.456 ± 0.072	0.421 ± 0.090	0.607 ± 0.088	0.602 ± 0.078	-	-
	SKIN	-	0.440 ± 0.098	0.450 ± 0.115	0.639 ± 0.133	0.709 ± 0.151	-	-
Lapses of attention	HYP	-	3 ± 2	1 ± 1	1 ± 1	2 ± 2	-	-
	SKIN	-	2 ± 1	1 ± 1	1 ± 2	3 ± 5	-	-
Accuracy (%)	HYP	-	96 ± 5	97 ± 3	96 ± 5	97 ± 3	-	-
	SKIN	-	98 ± 2	95 ± 5	98 ± 3	95 ± 9	-	-
Thermal sensation	HYP	4.1 ± 1.5	-	-	-	-	6.0 ± 0.8	8.3 ± 0.8
	SKIN	3.6 ± 0.8	-	-	-	-	6.7 ± 0.8	8.4 ± 0.5
Rating of perceive exertion	HYP	-	-	-	-	-	10.0 ± 3.0	18.7 ± 1.6
	SKIN	-	-	-	-	-	9.6 ± 3.0	19.2 ± 0.9
△Salivary cortisol concentration (%)	HYP	100 ± 0	-	-	-	-	74.2 ± 49.6	148.2 ± 82.5
	SKIN	100 ± 0	-	-	-	-	89.0 ± 61.3	122.0 ± 45.4
Heart rate (bpm)	HYP	64 ± 5	-	-	-	-	98 ± 18*	192 ± 8
	SKIN	69 ± 11	-	-	-	-	76 ± 11	189 ± 14

^*^p < 0.05, HYP vs. SKIN

### Heart rate

The HR was higher in the HYP trial than in the SKIN trial after the 45-min task (*p* = 0.021, *d* = 1.48), albeit there were no significant differences in HR before and after exercise between trials (Table 1; *p* > 0.05).

### Body water composition

There were no significant differences in USG both before (HYP: 1.019 ± 0.005, SKIN: 1.022 ± 0.009; *p* = 0.292, *d* = 0.41) and after (HYP: 1.016 ± 0.008, SKIN: 1.022 ± 0.009; *p* = 0.127, *d* = 0.71) trials. The TSL was higher in the HYP trial (1600 ± 306 g) than in the SKIN trial (770 ± 387 g; *p* = 0.001 *d* = 2.38). The dehydration level was higher in the HYP trial (2.37 ± 0.37%) than in the SKIN trial (1.14 ± 0.52%; *p* = 0.001, *d* = 2.73).

## Discussion

This study compared the effects of exposure to heat stress (high core and skin temperatures: HYP vs. high skin temperature alone: SKIN) in addition to a mental fatigue on aerobic exercise capacity in the heat. In accordance with the proposed hypothesis, the aerobic exercise capacity was impaired when combined with high core and skin temperatures (HYP) compared with high skin temperature only (SKIN).

Previous studies have investigated the combined effects of mental fatigue from a prolonged cognitive task and exposure to heat stress using protocol induced to the elevation of both Tc and Tsk [15, 16]. However, some exposures to heat stress (35–36℃ and 60%RH) at rest for approximately 90–120 min increase Tsk [33] but not Tc [34]. Therefore, this study employed protocol with (hyperthermia: HYP) and without (SKIN) Tc increase in addition to elevation of Tsk and mental fatigue. The results of this study are consistent with previous studies showing that reaching a high Tc impaired aerobic exercise capacity [3, 4], although mental fatigue is added. Therefore, when considering heat stress exposure in addition to mental fatigue, it is suggested that hyperthermia, which is accompanied by an increase in Tc more than an increase in Tsk alone, is additively induced by an impairment of aerobic exercise capacity. On the other hand, this observation suggested interaction effects between heat stress and mental fatigue [15], which followed the recently proposed “most severe strain takes precedence” principle [35], whereby the more severe strain, “heat stress”, takes precedence over the less severe strain “mental fatigue” [36]. Moreover, Van Cutsem et al. [16] suggested that stressing the brain (e.g., heat stress and mental fatigue) will impair endurance performance; however, at some point, further stressing the brain (e.g., combining heat stress and mental fatigue) will not result in a further decrease in endurance and cognitive performance, and a floor effect is observed. It is possible that a higher Tc was a more severe stressor than other stressors (i.e. mental fatigue) in this study, which, in turn, affected aerobic exercise capacity.

In the HYP trial, Tsk increased progressively during the 45-min task but decreased during exercise, which in turn greater the core-to-skin temperature gradient. In this study, the water temperature used in the HYP trial was 40℃, the same as in the previous study [15] and hotter than the chamber temperature. A possible explanation is that this phenomenon may have been caused by the drop in ambient temperature associated with entering the chamber after warm water immersion. In addition, the decrease in Tsk after immersion may be the result of heat transfer via evaporation because sweat is not able to evaporate during water immersion, and this result seems to be related to TS as well. It is known that a greater core-to-skin temperature gradient elicited by reducing Tsk improves cardiovascular load and prevents deterioration of oxygen supply to exercising muscles and brain, which in turn has a positive impact on exercise performance [37, 38]. However, even though the core-to-skin temperature gradient increased during exercise after warm water immersion, it did not affect the HR response and exercise time was shorter compared with exposure to the heat chamber. Therefore, this study suggested that the attainment of a high Tc appears to have been a limiting factor inhibiting aerobic exercise capacity rather than the magnitude of the core-to-skin temperature gradient, although there is no single Tc value associated with fatigue [39].

The state of mental fatigue induced by prolonged cognitive tasks could increase the perception of effort (RPE) [8, 10] and, therefore, reduce exercise capacity, since RPE has been deemed a key variable for stopping exercise [9]. However, changes in cortisol and the magnitude of mental fatigue caused by the 45-min task were similar levels between trials. Research on the difference between environmental conditions to load cognitive tasks is limited, but Valenza et al. [36] and Otani et al. [15] demonstrated that there were no significant differences in the magnitude of mental fatigue when the effects of cognitive tasks were examined under thermoneutral and hot environments. These results and the findings of this study indicate that mental fatigue may not be exacerbated by ambient temperature or an increase in Tc due to heat exposure. In addition, in hot environments, the limiting factor appears to be a higher Tc, and it has been suggested that an elevated Tc reduces the drive to exercise, which is reflected in higher RPE [40]. Conversely, RPE in this study was inconsistent with these above studies, and similar in value between the trials despite hyperthermia via warm water immersion before exercise. Schlader et al. [41, 42] reported that a change in thermal sensation and discomfort, and degree of Tsk affected voluntary exercise intensity, and, thus, RPE. The same value of perceptual discomfort and Tsk may have affected RPE in both trials in this study. The increase in RPE due to perceptual discomfort and decrease in Tsk can be improved by body cooling [43, 44], and further study is warranted to examine the effects of body cooling for combined mental load and heat exposure.

### Limitations

In the HYP trial, the participants were immersed in warm water, in contrast to the SKIN trial. It is established that hydrostatic pressure induces bradycardia and increases stroke volume during water immersion [45]. The HR in the HYP trial was observed to be higher than that in the SKIN trial following the completion of the 45-min task. Given the shallow water depth employed in this study and the elevated HR observed, it can be reasonably inferred that the effects of thermal stress and mental tasks due to water temperature are indeed significant.

In this study, it is possible that dehydration caused by warm water immersion affected aerobic exercise capacity in the HYP trial. Dehydration > 2% BM degrades aerobic exercise and cognitive/mental performance in hot environments [25]. In contrast, previous systematic reviews reported that trained athletes demonstrated a lower in decrease effects on performance than untrained individuals [46]. Trangmar & Gonzalez-Alonso [47] additionally indicated that dehydration amounting to 3–4%BM augments the development of physiological strain to the body, in turn reducing endurance exercise capacity. In the HYP trial, the dehydration level was 2.37 ± 0.37%, but since they did not exceed 3% and were highly trained athletes, we believe that dehydration did not have that much of an impact on their performance.

In previous studies [8, 16], the participants were given incentives to ensure high motivation levels during the experimental trials. However, the present study did not provide any motivational intervention to address heat-related illnesses resulting from excessive heat strain caused by overexertion. It was observed that one of the participants experienced heat-related illnesses during the warm water immersion in the HYP trial. To improve future exercise performance and reduce the impact of heat strain, it would be beneficial for upcoming research to consider motivational intervention, even when exercising in a hot environment.

## Conclusions

An elevated core body temperature, combined with increased skin temperature and mental fatigue, limits the aerobic exercise capacity of highly trained athletes in hot environments compared to heat exposure without an elevation of core body temperature. This suggests that athletes who are mentally fatigued should avoid conditions that increase their core body temperature to levels equivalent to hyperthermia before exercising to attenuate impairment in their endurance exercise performance in hot environments.

## Data Availability

Not applicable.

## Abbreviations

AX-CPT: AX-Continuous Performance Task
BM: Body mass
HR: Heart rate
HYP: Hyperthermia
V(●)O2max: Maximal oxygen uptake
M-VAS: Mental fatigue visual analog scale
RH: Relative humidity
RPE: Rating of perceived exertion
SKIN: Elevation in skin temperature
Tc: Body core temperature
TC: Thermal comfort
Tre: Rectal temperature
TS: Thermal sensation
TSL: Total sweating loss
Tsk: Mean skin temperature
TTE: Time to exhaustion
USG: Urine specific gravity
